# The Association Between Cholecystectomy and Colorectal Cancer in the Female Gender

**DOI:** 10.7759/cureus.20113

**Published:** 2021-12-02

**Authors:** Fahad Aurif, Harsimran Kaur, Jeoffrey Patrick G Chio, Mahdi Kittaneh, Bilal Haider Malik

**Affiliations:** 1 General Surgery, California Institute of Behavioral Neuroscience and Psychology, Fairfield, USA; 2 Family Medicine, California Institute of Behavioral Neurosciences and Psychology, Fairfield, USA; 3 General and Laparoscopic Surgery, California Institute of Behavioral Neuroscience and Psychology, Fairfield, USA; 4 Internal Medicine, California Institute of Behavioral Neurosciences and Psychology, Fairfield, USA

**Keywords:** cholecystectomy, colo rectal cancer, female gender, bile acids, oestrogen, incidence

## Abstract

Colorectal carcinoma (CRC) has been of great interest among researchers, and multiple causes have been proposed and accepted; however, cholecystectomy (CMY) as a potential cause for CRC, particularly in the female gender has not been studied in detail, despite multiple evidence suggesting a positive association. This review is directed at investigating the association between CMY and CRC in the female gender and aims at finding a potential cause for this association.

CRC involves cancer of the sigmoid and rectum. The composition of the bile acids is altered in patients after CMY, and the resultant secondary bile acids (BA) without a functioning gall bladder are exposed directly to the intestines, which could lead to cancer. An increase in fecal secondary bile acids is also described as high in the CMY population and has been linked to cancer. Right-sided GI cancers were attributed to CMY, although many earlier studies did not find this to be true. It is interesting to note a strong association between CRC and CMY in the female western population.

## Introduction and background

The incidence of colorectal cancer (CRC) is alarmingly high. There are greater than one to two million newly diagnosed CRC patients every year, with greater than 600,000 dying from the disease [1]. CRC is 9% of all cancer incidence [2-4]. It is the third most common malignancy and ranked fourth among the leading cause of cancer-related deaths worldwide [3]. The incidence of CRC in men worldwide is 9.4%, and 10.1% in women [4]. CRC has a variable geographic difference, more in the western world [2,4]. The incidence varies from greater than 40% per 100,000 people in the USA, Australia, New Zealand, and West Europe to less than 5% per 100,00 in Africa and Asia [2,3].

Multiple aetiologies have been proposed in the pathogenesis of CRC; common etiologies are age, inflammatory bowel disease, adenomatous polyps, strong family history, familial adenomatous polyposis, hereditary non-polyposis colon cancer, diets high in animal fat, smoking, and alcohol consumption [2]. In this article, cholecystectomy (CMY) as a potential cause for CRC is investigated, particularly in the female sex, as there is some evidence to suggest that there may be an association between the two and for the fact that female sex is an independent risk factor for the development of gall stone disease [5]. CMY has traditionally been accepted as the gold standard for gallstone disease and is one of the most common operations done the world over. The female gender is regarded as one of the risk factors for gallstone disease [5]. The worldwide prevalence of CMY is studied in detail; however, there are sparse data on the incidence of CMY.

The association between the two has been carefully studied in multiple papers, and some biological mechanisms involving the GI tract have been hypothesized, of which the usually accepted theory is bile acids (BA), synthesized in the liver, and stored in the gall bladder, as a potential carcinogen for CRC [6-10]. After a CMY, the exposure of these bile acids to the intestinal mucosa is increased, and CMY causes negative feedback to the synthesis of bile acids in the liver [10,11]. The question, if this association is higher in the female gender, arises simply because the female gender is itself a risk for gallstone disease, which could lead to surgical intervention.

We intend to study this association, with particular reference to the most common site, time interval to diagnosis, the subtype of CRC, and the possible role of estrogen in this association, and whether all female patients undergoing a CMY will have to be closely monitored for CRC.

## Review

Several studies have tried to find a link between CRC and CMY; some have found a possible association, and some have had conflicting results. 

The pathophysiology behind the association of colorectal cancer and cholecystectomy

It has been well established that genetic mutations, polyps, adenomas, and the constant effects of anaerobic bacteria like clostridium on the intestinal mucosa play a major role in the pathogenesis of CRC [8]. The gall bladder is assigned the physiological function to store and concentrate the BA [9], which acts as a buffer for the direct effect of the BA on the intestinal mucosa. The incidence of gallstone disease is increased [10], and CMY plays an important role in treatment, causing the physiological properties of bile acids to possibly change postoperatively. Following a CMY, the bile acids drain continuously into the intestines due to the absence of bile storage and lack of the relaxation of the sphincter of Oddi, as was demonstrated in many experimental studies [11]. Also, there is a change in the composition and secretion of bile acids [11]. The intestinal mucosa is continuously stimulated by secondary bile acids, which are metabolites of the bile acids [12]. The products of the intestinal microflora, due to continuous stimulation by the bile acids, along with secondary bile acids elevate the risk for CRC in patients following a CMY [13].

Secondary BA particularly lithocholic acid (LCA) and deoxycholic acid (DCA) has a similar molecular structure to carcinogenic polycyclic aromatic hydrocarbons, hence LCA and DCA are considered carcinogenic in CRC [13]. There are multiple biochemical and physiological aspects of secondary BA that play an essential role in the pathogenesis of CRC [14]. The increased physiological levels of BA and secondary bile acids lead to Apoptosis resistance, genomic instability, and BA hydrophobicity which are linked to malignancy [15,16]. These aggravate colonic polyps arising from the glandular epithelium, which leads to CRC [17]. Furthermore, in patients with CRC, there is an escalation in the dehydrogenase activity of bacterial beta-glucuronidase, alpha decarboxylase, and cholesterol [18]. 7-Alpha decarboxylase converts the BA to deoxycholic acid, which is a potential carcinogen [19].

Nagathihalli et al., in their study, show that BA induces tumorigenesis by stimulating mitogenic receptor tyrosine kinase signaling pathways and propose a mechanism involving ectodomain shedding of the epidermal growth factor receptor ligands amphiregulin (AREG), and prove the role of AREG in CRC [8]. Zuccato et al, in their study, also associate CRC with CMY; they reason the metabolic activity of the colonic flora after CMY, a possible cause [7,13]. Their study group had higher lithocholic (LCA), chenodeoxycholic acid, and LCA/deoxycholic acid concentrations in stools than their control, which was similar to patients with CRC. 

**Figure 1:**
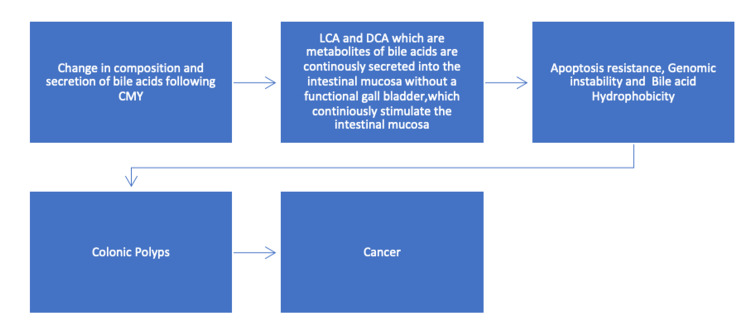
The flowchart is describing the etiology and pathophysiology of CRC after CMY. Following a CMY there is a change in the composition and secretion of bile acids, the metabolites of bile acids namely lithocholic acid and deoxycholic acid are secreted directly into the intestinal mucosa without a functional gall bladder, which in turn stimulate the intestinal mucosa leading to multiple cellular phenomena called apoptosis resistance, genomic instability, and bile acids hydrophobicity. These phenomena can cause colonic polyps over time, leading to cancer. LCA: lithocholic acid, DCA: deoxycholic acid, CRC: colorectal cancer, CMY: cholecystectomy, BA: bile acids.

The etiology and pathophysiology of CRC after CMY is described below in Figure 1.

Cholecystectomy and right-sided colon cancer

An association between gallstones/cholecystectomy and right-sided colon cancer has been debated since 1982 [20]. There have been multiple cohort studies linking both conditions [21-27]; one of the studies has been successful in proving an association between gall stones without a CMY and right-sided colon cancer [24]. Among the many possible reasons for such an association is higher proximal colonic absorption of fecal secondary bile acids [28], proving as carcinogens [29]. Furthermore, CMY alters the bacterial composition of the colon, with phylum Bacteroidetes species playing a major role in the pathogenesis of CRC [30]. Sessile serrated adenomas, considered premalignant, are commonly found in the right colon at colonoscopy. The mechanism of malignant transformation is explained by the serrated neoplastic pathway, which includes a mutation in the oncogene BRAF, microsatellite instability, mutations in the CpG island methylator phenotype [31]. Sessile serrated adenomas are associated with multiple genetic mutations resulting in oncogene-induced senescence. These mechanisms are accelerated by fecal secondary Bile acids [32]. Many researchers from multiple centers have studied this extensively and have arrived at different conclusions.

A handful of case-control studies have demonstrated an association between colon cancer and CRC, without mention of the site and gall stones [33]; however, some studies fail to associate between CMY and CRC [34] and describe certain confounding factors such as dietary habits popular in the west as being rich in fats, which in turn can cause increased fecal secondary BA [35]. Biases of many orders have been used as criticism to studies associating the two and have called for the need for further prospective studies as needed to find an association between asymptomatic gallstone disease and right-sided colon cancers. Lagergren et al. identified post CMY patients through the Swedish inpatient register from 1965 to 1997, and were followed up for CRC, and concluded that CMY does increase the risk for the development of adenocarcinoma and carcinoids in the small bowel and right-sided colon cancers, interestingly, he did not find any association with CMY to distal colon or rectal cancers [23]. This positive correlation might be due to the natural tendency of those patients for colon cancers or maybe some genetic mutations already present. Nogueira et al. concluded in 2014 that CMY is linked to cancers throughout the GI tract, particularly gastric carcinoma, excluding the cardiac, small bowel carcinoids, liver, and pancreatic cancers; he also notes CRC risk decreased with increasing distance from the biliary tract [25].

No definite conclusions can be drawn to associating exclusive right-sided colon cancers to CMY, as the studies showing a positive correlation needs more information about, how unbiased and authentic these studies are.

Colorectal cancer and cholecystectomy in the female gender

Colorectal cancer involves cancer of the large intestine, which includes the sigmoid colon and rectum. This has been an area of great interest among researchers, and many studies supporting and conflicting with an association have been published and debated. The vast majority of the studies could not find an association between CRC and CMY [36-40], although some papers suggest twofold risks 10-20 years after a CMY. The risk of cancer is described to decrease as the distance from the biliary duct increases [36], and hence, rectal cancers have no association with CMY. However, a recent meta-analysis of 10 cohort studies conducted by Zhang et al. concludes an increased risk for CRC after CMY in the western population, particularly in the female sex [19]. Schernhammer et al., in their observation, have also proposed significant CRC risk in patients with cholecystectomy [41]. A potential cause for this association in the female sex is yet unknown and poorly understood. Gallstone disease is more prevalent in the female sex [4]. We hypothesize that CMY is performed more frequently in the feminine gender than the male gender. No studies were found to equate this association to the female gender.

Cholesterol is thought to play a significant role in the understanding of this association. Cholesterol is metabolized in the liver cells to form BAs. The synthesis of HGM-CoA Reductase in the liver is induced by insulin, which in turn increases the synthesis of cholesterol. High physiological levels of BA occur, which can cause insulin resistance, hyperinsulinemia, and obesity; this could explain the high incidence of CRC following CMY in the western world. The primary female sex hormone is oestradiol, which is a precursor to cholesterol, that affects the synthesis of bile in the liver cells, a possible explanation for increased risk in the female sex.

Table 1 describes some of the recent articles and their conclusions.

**Table 1:** Recently published articles on the association of CRC with CMY. CRC: colorectal carcinoma, CMY: cholecystectomy.

Authors	Year of study	Country of origin	Salient features
Zhang et al. [19]	2018	China	CMY can increase the risk of CRC in the female gender.
Shang et al. [37]	2016	Australia	CMY is not at all a risk for CRC.
Coats and Shimi [34]	2015	Dundee, Scotland	No clear association between CMY and CRC could be established.
Chen et al. [27]	2014	Taiwan	Gall stone disease patients have a high risk of GI cancer; the risk is elevated after CMY within the first five years.
Goldacre et al. [40]	2012	UK	Intestinal cancers are associated with gall stones; the risk is elevated after CMY.
Schernhammer et al. [41]	2003	USA	CMY increases the risk of CRC, after adjustments of other CRC risk factors.

The metanalysis by Zhang et al., where he carefully studies as many as 10 cohort studies, clearly concludes a high association between CMY and CRC in the western female gender [19], and most other studies linking CMY to CRC cannot prove an association and high incidence in the female sex. However, they all confirm this association as more elevated in the western population. Only further studies can conclusively prove this trend, with particular regard to the high incidence of CRC in the western female population. The possibility of a gene responsible for this association in the female sex is a gray area and needs to be studied extensively.

The role of estrogen in colorectal carcinogenesis

Estrogens are steroid hormones that are derived from cholesterol. They are produced by the aromatization of androgens, mostly in the ovary and also other tissues like muscle, adipose, and nervous tissue. Estrogens include estrone, estriol, and 17β-oestradiol (the biologically active metabolite of estrogen). They are attributed to controlling sexual behavior and reproductive function in women; they play an essential role in the homeostasis of cardiovascular, nervous, immune systems, and bone metabolism [42]. Estrogens are not just linked to cancers of the female reproductive system but also cancers of the lung and the gastrointestinal system. To understand the effects of estrogen in CRC, a basic understanding of the structure, function, and mechanism of action of estrogen receptors is essential.

Estrogen receptors are composed of modular proteins that are organized in functional domains (N-terminal domain, DNA-binding domain, hinge region, ligand-binding C-terminal domain) that are designed to mediate ligand-dependent gene expression [43]. Estrogens bind to two different estrogen receptors, namely ESR 1(ER-alpha or ERα) and ESR 2(ER-beta or ERβ). ERα and ERβ are encoded by two separate genes ESR 1 and ESR 2; these genes are located on different chromosomes and share the same sequence homology concerning the DNA-binding region (97% homology) and to a lesser extent in the ligand-binding area (59% homology). This explains the shared mechanism of action with differences in their specificities and sensitivities for different ligands. Estrogen receptor ERβ is expressed predominantly in both normal as well as malignant colonic epithelium and no or limited expression of ERα in the colon [44]. It is interesting to note that the expression of ERβ is reduced in CRC tumorigenesis [45], and many studies have described an inverse relationship between ERβ expression and tumor progression [46]. In essence, estrogen-mediated signaling may be protective of CRC.

**Figure 2:**
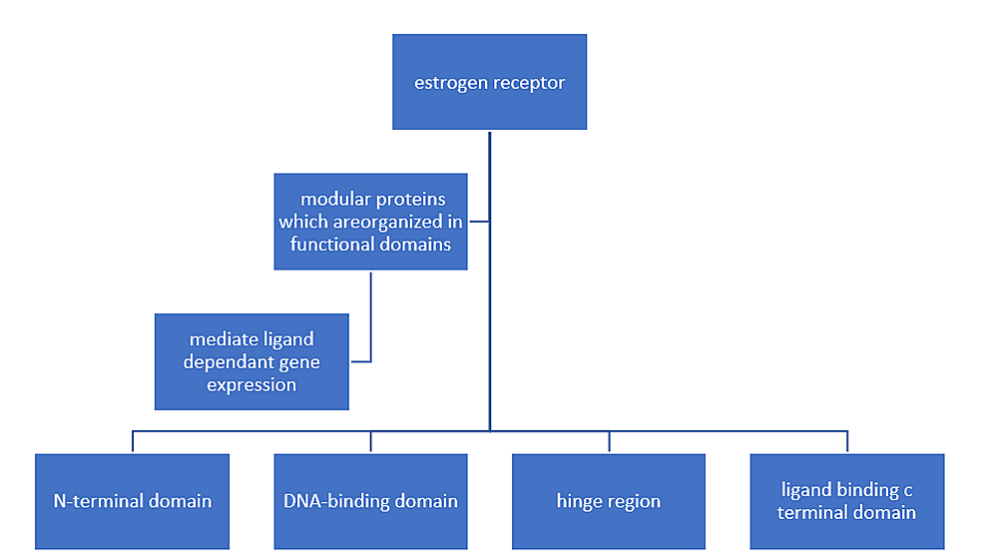
Functional domains of estrogen receptors.

Functional domains of estrogen receptors are shown in Figure 2.

**Figure 3:**
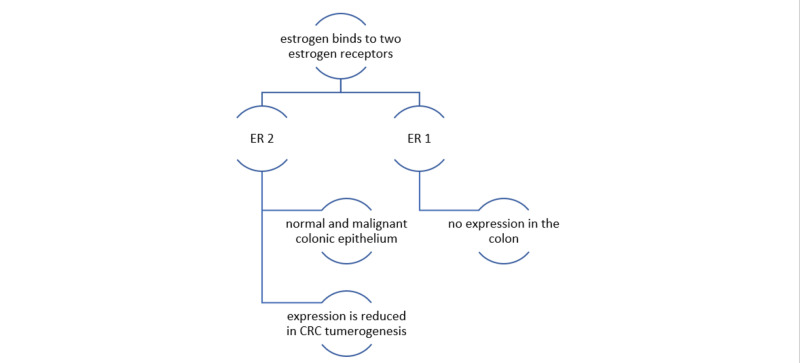
Expression of estrogen receptors in CRC tumorigenesis: estrogen binds to two receptors, ERα and ERβ; they are encoded by two separate genes ESR 1 and ESR 2. Estrogen receptor ERβ is expressed predominantly in both normal as well as malignant colonic epithelium and no or limited expression of ERα in the colon. The expression of ERβ is reduced in CRC tumorigenesis. Studies have described an inverse relationship between ERβ expression and tumor progression. IN essence, estrogen-mediated signaling may be protective of CRC. ESR 1, ERα (estrogen receptors one or estrogen receptors alpha); ESR 2, ERβ (estrogen receptors two or estrogen receptors beta).

Figure 3 describes the expression of estrogen receptors in colorectal cancer tumorigenesis.

It would be worthwhile to study further on this phenomenon to find if this can explain the high association of CRC with CMY in the female sex, on the lines of whether a CMY and secondary BA have interference in the protective effect of estrogen-mediated signals to CRC.

## Conclusions

This study was done with the intent to generate an interest concerning an association between CMY and CRC in the female gender, with a focus on the most common site in the colon, the time interval for cancer to occur after CMY, the subtype of CRC involved, a possible role of estrogen in this association and most importantly, if it is prudent to closely monitor all female patients undergoing a CMY, for CRC. We studied this association in the feminine gender as the female gender is in itself a risk factor for gallstone disease, and we hypothesized that CMY is higher in the female gender. We could conclude that CRC after CMY is real and has much to do with the composition of bile acids that are altered without a functional gall bladder, and the resultant secondary bile acids are secreted directly into the intestines, which elevates the risk for CRC. As for the site for cancer, studies are associating the entire GI tract, including stomach cancers excluding the cardiac, small bowel carcinoids, hepatopancreatobiliary, and right-sided colon cancers. Many recent studies have evidence to suggest a high incidence of CRC after CMY in the western female population. Most studies do not describe the subtype of CRC or the time interval to diagnosis, although some reviews mention 10 to 20 years following a CMY. Estrogen was found to be protective against CRC via the estrogen-mediated signaling pathway. The importance of this study lies in understanding the etiopathology behind the development of CRC after CMY, and also describes this association as higher in the female western population. The review is essential to answer precise questions on why this association is higher in the feminine gender and to prompt further studies in this direction.

We would recommend further prospective randomized controlled trials on the association between CMY and CRC, particularly in the female western population, and also to explore this association with larger sample size and over longer follow-up periods.

## References

[1] Conrad SJ, Essani K (2014). Oncoselectivity in oncolytic viruses against colorectal cancer. J Cancer Therapy.

[2] Haggar FA, Boushey RP (2009). Colorectal cancer epidemiology: incidence, mortality, survival, and risk factors. Clin Colon Rectal Surg.

[3] Wiseman M, Cannon G, Butrum R, Martin G (2021). Food, nutrition, physical activity, and the prevention of cancer: a global perspective summary. http://www.ph.ucla.edu/epi/faculty/zhang/courses/epi244/readings/ref%204-1.pdf.

[4] Boyle P, Langman JS (2000). ABC of colorectal cancer: epidemiology. BMJ.

[5] Novacek G (2006). Gender and gallstone disease. Wien Med Wochenschr.

[6] Elizabeth TF (2008). Cancer epidemiology and prevention. Am J Epidemiol.

[7] Zuccato E, Venturi M, Di Leo G, Colombo L, Bertolo C, Doldi SB, Mussini E (1993). Role of bile acids and metabolic activity of colonic bacteria in increased risk of colon cancer after cholecystectomy. Dig Dis Sci.

[8] Nagathihalli NS, Beesetty Y, Lee W, Washington MK, Chen X, Lockhart AC, Merchant NB (2014). Novel mechanistic insights into ectodomain shedding of EGFR Ligands Amphiregulin and TGF-α: impact on gastrointestinal cancers driven by secondary bile acids. Cancer Res.

[9] Turumin JL, Shanturov VA, Turumina HE (2013). The role of the gallbladder in humans. Rev Gastroenterol Mex.

[10] Goral V (2016). Gallstone etiopathogenesis, Lith and Mucin genes and new treatment approaches. Asian Pac J Cancer Prev.

[11] Koga S, Kaibara N, Takeda R (1982). Effect of bile acids on 1,2-dimethylhydrazine-induced colon cancer in rats. Cancer.

[12] Berr F, Stellaard F, Pratschke E, Paumgartner G (1989). Effects of cholecystectomy on the kinetics of primary and secondary bile acids. J Clin Invest.

[13] Hill M J (1990). Bile flow and colon cancer. Mutation research.

[14] Bernstein H, Bernstein C, Payne CM, Dvorak K (2009). Bile acids as endogenous etiologic agents in gastrointestinal cancer. World J Gastroenterol.

[15] Payne CM, Bernstein C, Dvorak K, Bernstein H (2008). Hydrophobic bile acids, genomic instability, Darwinian selection, and colon carcinogenesis. Clin Exp Gastroenterol.

[16] Powell AA, LaRue JM, Batta AK, Martinez JD (2001). Bile acid hydrophobicity is correlated with induction of apoptosis and/or growth arrest in HCT116 cells. Biochem J.

[17] Zhao MF, Huang P, Ge CL, Sun T, Ma ZG, Ye FF (2016). Conjugated bile acids in gallbladder bile and serum as potential biomarkers for cholesterol polyps and adenomatous polyps. Int J Biol Markers.

[18] Selmin OI, Fang C, Lyon AM (2016). Inactivation of adenomatous polyposis coli reduces bile acid/farnesoid X receptor expression through Fxr gene CpG methylation in mouse colon tumors and human colon cancer cells. J Nutr.

[19] Zhang Y, Liu H, Li L (2018). Correction: Cholecystectomy can increase the risk of colorectal cancer: a meta-analysis of 10 cohort studies. PLoS One.

[20] Maringhini A, Maringhini M (2017). Gallstones and colon cancer: a result of a wrong study revived. Gastroenterology.

[21] Johansen C, Chow WH, Jørgensen T, Mellemkjaer L, Engholm G, Olsen JH (1996). Risk of colorectal cancer and other cancers in patients with gall stones. Gut.

[22] Goldacre MJ, Wotton CJ, Abisgold J, Yeates DG, Collins J (2012). Association between cholecystectomy and intestinal cancer: a national record linkage study. Ann Surg.

[23] Lagergren J, Ye W, Ekbom A (2001). Intestinal cancer after cholecystectomy: is bile involved in carcinogenesis?. Gastroenterology.

[24] Theresa S, Xiao YY (2005). Cholecystectomy and the risk of colorectal cancer. Am J Gastroenterol.

[25] Nogueira L, Freedman ND, Engels EA, Warren JL, Castro F, Koshiol J (2014). Gallstones, cholecystectomy, and risk of digestive system cancers. Am J Epidemiol.

[26] Vinikoor LC, Robertson DJ, Baron JA, Silverman WB, Sandler RS (2007). Cholecystectomy and the risk of recurrent colorectal adenomas. Cancer Epidemiol Biomarkers Prev.

[27] Chen YK, Yeh JH, Lin CL, Peng CL, Sung FC, Hwang IM, Kao CH (2014). Cancer risk in patients with cholelithiasis and after cholecystectomy: a nationwide cohort study. J Gastroenterol.

[28] Linos D, Beard CM, O'Fallon WM, Dockerty MB, Beart RW Jr, Kurland LT (1981). Cholecystectomy and carcinoma of the colon. Lancet.

[29] Hill MJ, Drasar BS, Williams RE (1975). Faecal bile-acids and clostridia in patients with cancer of the large bowel. Lancet.

[30] Keren N, Konikoff FM, Paitan Y, Gabay G, Reshef L, Naftali T, Gophna U (2015). Interactions between the intestinal microbiota and bile acids in gallstones patients. Environ Microbiol Rep.

[31] Leggett B, Whitehall V (2010). Role of the serrated pathway in colorectal cancer pathogenesis. Gastroenterology.

[32] Gala MK, Mizukami Y, Le LP (2014). Germline mutations in oncogene-induced senescence pathways are associated with multiple sessile serrated adenomas. Gastroenterology.

[33] Gafà M, Sarli L, Sansebastiano G, Lupi M, Longinotti E, Rigamonti PP, Peracchia A (1987). Gallstones and risk of colonic cancer: a matched case-control study. Int Surg.

[34] Coats M, Shimi SM (2015). Cholecystectomy and the risk of alimentary tract cancers: a systematic review. World J Gastroenterol.

[35] Reddy BS, Wynder EL (1973). Large-bowel carcinogenesis: fecal constituents of populations with diverse incidence rates of colon cancer. J Natl Cancer Inst.

[36] Ekbom A, Yuen J, Adami HO, McLaughlin JK, Chow WH, Persson I, Fraumeni JF Jr (1993). Cholecystectomy and colorectal cancer. Gastroenterology.

[37] Shang J, Reece JC, Buchanan DD (2016). Cholecystectomy and the risk of colorectal cancer by tumor mismatch repair deficiency status. Int J Colorectal Dis.

[38] Nielsen GP, Theodors A, Tulinius H, Sigvaldason H (1991). Cholecystectomy and colorectal carcinoma: a total-population historical prospective study. Am J Gastroenterol.

[39] Adami HO, Krusemo UB, Meirik O (1987). Unaltered risk of colorectal cancer within 14-17 years of cholecystectomy: updating of a population-based cohort study. Br J Surg.

[40] Goldacre MJ, Abisgold JD, Seagroatt V, Yeates D (2005). Cancer after cholecystectomy: record-linkage cohort study. Br J Cancer.

[41] Schernhammer ES, Leitzmann MF, Michaud DS, Speizer FE, Giovannucci E, Colditz GA, Fuchs CS (2003). Cholecystectomy and the risk for developing colorectal cancer and distal colorectal adenomas. Br J Cancer.

[42] Gruber CJ, Tschugguel W, Schneeberger C, Huber JC (2002). Production and actions of estrogens. N Engl J Med.

[43] Ascenzi P, Bocedi A, Marino M (2006). Structure-function relationship of estrogen receptor alpha and beta: impact on human health. Mol Aspects Med.

[44] Thomas C, Gustafsson JÅ (2011). The different roles of ER subtypes in cancer biology and therapy. Nat Rev Cancer.

[45] Rudolph A, Toth C, Hoffmeister M (2012). Expression of oestrogen receptor β and prognosis of colorectal cancer. Br J Cancer.

[46] Caiazza F, Ryan EJ, Doherty G, Winter DC, Sheahan K (2015). Estrogen receptors and their implications in colorectal carcinogenesis. Front Oncol.

